# BuFeiXiaoJiYin ameliorates the NLRP3 inflammation response and gut microbiota in mice with lung cancer companied with Qi-yin deficiency

**DOI:** 10.1186/s12935-022-02543-9

**Published:** 2022-03-15

**Authors:** Rui-yuan Jiang, Ting Wang, Qiao-yu Lan, Yan-chun Qin, Ting-ting Man, Hua Sun, Zhu-long Li, Xiao-ting Zhong, Chun-mei Mo, Zhen Rong

**Affiliations:** 1 Department of Graduate Student, Guangxi University of Chinese Medicine, No. 13, Wuhe Road, Qingxiu District, Nanning, 530022 Guangxi China; 2 Department of Dean’s Office, Guangxi University of Chinese Medicine, No. 13, Wuhe Road, Qingxiu District, Nanning, 530022 Guangxi China; 3 Department of Graduate Student, Guangzhou Medical University, No. 1, Xinzao Road, Fanyu District, Guangzhou, 511495 Guangdong China; 4 Department of Hepatology, Bao’an Authentic TCM Therapy Hospital, No. 99, Laian Road, Baoan District, Shenzhen, 518100 Guangdong China; 5 Department of Oncology, Bao’an Authentic TCM Therapy Hospital, No. 99, Laian Road, Baoan District, Shenzhen, 518100 Guangdong China

**Keywords:** Lung cancer, Qi-yin deficiency, NLRP3 inflammation response, Gut microbiota, BuFeiXiaoJiYin

## Abstract

**Introduction:**

NLRP3 inflammasome responses and gut microbiota have been shown an important role in lung cancer, however, the relationship between gut microbiota and NLRP3 inflammasome responses in lung cancer with Qi-yin deficiency remains elusive.

**Methods:**

To investigate the effect of the traditional Chinese medicine BuFeiXiaoJiYin (BFXJY) on NLRP3 inflammasome responses and dysbiosis in lung cancer with Qi-yin deficiency, the female BALB/cA-nu mice were treated with LPS and ATP to induce inflammation, and were intragastrically treated with warm Chinese medicine and smoked with shavings to induce Qi-yin deficiency, as well as were injected with 1 × 10^7^/ml A549 cells to simulate lung cancer. Then the three different doses of BuFeiXiaoJiYin (BFXJY) and positive control (CRID3) were used for intervention in mice for 27 consecutive days. Then, we estimated the protection effect of BFXJY on lung cancer mice with Qi-yin deficiency, through deterring tumor growth, NLRP3 inflammasome, PKC signaling, and homeostasis of gut microbiota.

**Results:**

In this study, we found that BFXJY could inhibit the tumor growth in lung cancer with Qi-yin deficiency by reducing the production of IL-1β and IL-18 and inhibiting NLRP3 inflammasome activation, which might be associated with the inhibition of PKC signaling. Furthermore, BFXJY could promote microbial diversity and balance the microbial composition changes induced by inflammation and Qi-yin deficiency in lung cancer.

**Conclusion:**

BuFeiXiaoJiYin ameliorates the NLRP3 inflammation response and gut microbiota in mice with lung cancer companied with Qi-yin deficiency. Our study provides a theoretical basis for the clinical development of therapeutic drugs targeting to treat lung cancer.

## Introduction

Lung cancer is the dominating cause of death around the world [1]. The incidence and mortality of male lung cancer rank first among all malignant tumors, and that of female lung cancer ranks second [1, 2]. Therein, non‐small cell lung cancer (NSCLC) which accounts for 85% of lung cancers, is estimated to cause 1.76 million deaths in 2018 [3]. Especially, the early diagnosis rate of lung cancer is very low. Thus, most patients are in the advanced stage at the time of diagnosis and cannot be treated with surgery. In recent years, the application of Chinese medicine in malignant tumors has received widespread attention. Traditional Chinese medicine (TCM) believes that the etiology and pathogenesis of lung cancer can be attributed to the evil reality and the right deficiency. The lungs belong to the delicate organs with sober preference and are easy to heat and cold, therefore, the invasion of external evils must first invade the lungs, and the lung's Qi and Yin damage will bear the brunt. Consequently, Qi-yin deficiency in lung cancer dialectical classification is more common. Thus, it is an urgent requirement for establishing the potential pathogenic mechanism and effective therapy of lung cancer with Qi-yin deficiency.

As a core determinant of tumorigenesis, the inflammation affects cancer progression and development [4, 5]. Inflammasomes, especially the nucleotide-binding oligomerization domain (NOD)-like receptor family, pyrin domain-containing protein 3 (NLRP3) inflammasome, powerfully drive the recruitment of inflammatory cells and the modulation of immune responses in a variety of organs, including the lung [6]. NLRP3 inflammasome assembles with the adaptor, apoptosis-associated speck-like protein containing a caspase recruitment domain (also called ASC), that promotes the binding of the pyrin domain (PYD) at the N-terminus of NLRP3 and the CARD domain of the effector pro-caspase-1 to recruit NLRP3 [7]. NLRP3 inflammasome can be activated by a variety of stimuli, such as pathogen-associated molecular patterns (PAMPs) including viruses, bacteria, and fungi, as well as damage-associated molecular patterns (DAMPs) such as adenosine triphosphate (ATP) and beta-amyloid deposits [8]. After stimulated, the NLRP3 inflammasome can bind to the ASC, and then the CARD domain recruits the inactive pro-enzyme form (pro-caspase-1β) to form the inflammasome and activate the pro-caspase-1β to caspase-1. In turn, caspase-1 cleaves pro-IL-1β and pro-IL-18 into their active forms IL-1β and IL-18, respectively. The responses of IL-1β and IL-18 can trigger the recruitment of immune cells, activate alternative proinflammatory signaling pathways, and lead to cell damage and death [9]. After the NLRP3 inflammasome is activated, it cannot produce self-inhibition even in a resting state, which results in the continuous production of IL-1β and IL-18, finally causing long-term effects on inflammation and tumor diseases [10]. Hui Kong et al. [11] showed that the level of NLRP3 inflammasome, IL-1β, and IL-18 in lung cancer cells and tissues was significantly increased compared to that in normal lung cells and para-carcinoma tissues, which indicated that NLRP3 inflammasome mediated inflammatory response was involved in the progress of lung cancer. Moreover, a previous study also reported that the level of IL-1β, IL-6, and TNF-α was notably up-regulated in lung cancer with Qi-yin deficiency [12]. However, the role of NLRP3 inflammasome in lung cancer with Qi-yin deficiency still needs more attention.

It has been demonstrated that the gut microbiome can impact inflammation and disease outcomes via the modulation of NLRP3 inflammasome responses [13]. Over 1 × 10^14^ bacterial cells and 500 various species were harbored in the human gut [14], which exert multiple functions, such as immunological, metabolic, and protective roles [15]. Accumulating evidence indicates the alteration of microbiome composition was associated with the NLRP3 inflammasome responses. Mice treated with a broad-spectrum antibiotic cocktail showed the imbalances in microbiota composition and an increase of ileal protein expression of NLRP3, ASC, caspase-1, IL-1β, cleaved IL-1β, and IL-18 [16]. Another study revealed that bacterial load was reduced after mice were orally treated with an antibiotic cocktail, which was associated with the enhancement of serum IL-1β, cortical level of NLRP3 and ASC, and brain cortical and small intestinal level of IL-1β and IL-18 [17]. Moreover, dysbiosis is involved in an increase of a variety of diseases including various cancers. Fernández MF [18] and Raza MH [19] have been summarized the role of gut microbiota in the progression and development of different cancers including breast cancer, colorectal cancer (CRC), gall bladder cancer, mucosa-associated lymphoid tissue (MALT) lymphoma, ocular adnexa MALT cancer, ovarian carcinoma, oral cancer, and lung carcinoma. Furthermore, the relationship between gut microbiota and lung cancer was investigated both in the mouse model [20] and human beings [21]. However, the relationship between gut microbiota and NLRP3 inflammasome responses in lung cancer with Qi-yin deficiency remains elusive.

BuFeiXiaoJiYin (BFXJY) is a traditional Chinese medicine (TCM) compound that is created by our group based on many years of clinical experience. The prescription is composed of Astragalus, Ophiopogon japonicas, Aster, Thunberg fritillary bulb, Coltsfoot, Schisandra, Mulberry Bark, *Oldenlandia diffusa*, *Scutellaria barbata*, *Curcuma aeruginosa* Roxb., *Panax notoginseng*, and Turtle shell. In the prescription, Astragalus and Ophiopogon japonicas nourish yin and lungs; Aster, Thunberg fritillary bulb, Coltsfoot and Schisandra nourish the lungs, relieve phlegm, relieve cough, and dispel congestion; Mulberry Bark, *Oldenlandia diffusa*, and *Scutellaria barbata* clear the lungs and detoxify; *Curcuma aeruginosa* Roxb. And Turtle shell removes blood stasis, softens hard, and dispels knots; *Panax notoginseng* promotes blood circulation to stop bleeding. The whole prescription not only eliminates tumors and dispels masses, but also strengthens the body and cures the symptoms and root causes. Moreover, our previous studies [22, 23] have been shown good effects in the treatment of lung cancer, chemotherapy attenuation, and reverse transformation therapy resistance. Furthermore, Mulberry Bark and Curcuma zedoary have been exhibited a well inhibitory effect on the expression of IL-1β, IL-18, caspase-1, and NLRP3-ASC, which suggested that BFXJY can exert an anti-inflammation role through NLRP3 inflammasome responses [24]. Moreover, the gut microbiota is the focus of TCM treatment, because many effective components of TCM are produced with strong biological activity after the metabolism of gut microbiota, so BFXJY might play a therapeutic role through gut microbiota [25, 26].

Therefore, in the present study, we reported that BFXJY can alleviate NLRP3 inflammasome responses and regulate dysbiosis in lung cancer with Qi-yin deficiency in vivo. The results of this study will provide new sights and methods for the therapy of lung cancer with Qi-yin deficiency.

## Materials and methods

### Animal

Female BALB/c-nu mice (age: 5–6 weeks, weight: 18–22 g) were purchased and acclimated to SPF conditions for 7 days before experiments at the Animal Experiment Center of Guangxi Medical University. Mice were provided with a 12 h/12 h light–dark cycle and fed with a standard diet and water ad libitum at (25 ± 2) ℃, and 40–60% relative humidity. All the procedures were carried out strictly based on the National Institute of Health Guide for the Care and Use of Laboratory Animals. Also, the study was ratified by the Board and Ethics Committee of Guangxi Traditional Medicine University.

### Cell culture and drug administration

NSCLC line A549 was purchased from the cell bank of Peking University Cancer Hospital, and cultured in DMEM (Sigma, St. Louis, MO, USA) supplied with 10% FBS (Sigma) and 1% streptomycin and penicillin (Sigma) and maintained at 37 °C with 5% carbon dioxide (CO_2_). A549 cells were inoculated in 96-well plates with a density of 1 × 10^4^/well and then maintained for 24 h at 37 °C in 5% CO_2_. Subsequently, cells were treated with 1 μg/ml LPS for 6 h and then 5 μM ATP for 30 min to induce inflammation.

### Experimental groups and drug administration

40 mice were randomly divided into eight groups (n = 5), including control, Qi-yin deficiency, LPS-ATP, Qi-yin deficiency + LPS-ATP, Positive group, BFXJY low-dose group (Low-dose group), BFXJY middle-dose group (Middle-dose group), and BFXJY high-dose group (High-dose group). Mice in Qi-yin deficiency, Qi-yin deficiency + LPS-ATP, Positive group, Low-dose group, Middle-dose group, and High-dose group were built with Qi-yin deficiency model, as intragastrically treated with 20 g/kg warm Chinese medicine (9 g each of Chinese ephedra, *Aconitum carmichaeli* Debx, Asarum, and *Arisaema heterophyllum* Blume, Jiangyin Tianjiang Pharmaceutical Co., Ltd., Jiangsu, China) and smoked with shavings for 30 min once a day for 10 consecutive days. However, mice in control and LPS-ATP groups were intragastrically treated with the same volume of normal saline (Solarbio, Beijing, China) and not smoked with shavings once a day for 10 consecutive days. Then, 0.2 ml 1 × 10^7^ /ml A549 cells were injected into the right-back buttocks of mice in control and Qi-yin deficiency group, while 0.2 ml 1 × 10^7^ /ml A549 cells treated with LPS and ATP were injected into the right-back buttocks of mice in LPS-ATP, Qi-yin deficiency + LPS-ATP, Positive group, Low-dose group, Middle-dose group, and High-dose group. Next, drug intervention was given at 0, 3, 6, 9, 12, 15, 18, 21, 24, and 27 days after xenograft assay. Mice in Positive group, Low-dose group, Middle-dose group, and High-dose groups were treated with 200 μl 1 mg/kg CRID3 (Amgen, California, USA), 500 μl 2.5 g/kg BFXJY (Jiangyin Tianjiang Pharmaceutical Co., Ltd., main contents: scutellarin, concentration is 0.15 ~ 0.22 mg/g), 500 μl 5.0 g/kg BFXJY and 500 μl 10.0 g/kg BFXJY respectively, while mice in control, Qi-yin deficiency, LPS-ATP and Qi-yin deficiency + LPS-ATP groups were all treated with 500 μl normal saline. The tumor size of each group model at 0, 3, 6, 9, 12, 15, 18, 21, 24, 27 days was measured, and all mice were sacrificed for experiments at day 27. After the mice were intraperitoneally anesthetized with sodium pentobarbital (40 mg/kg), abdominal aorta blood was taken, and serum was isolated and stored at − 80 °C for further assays. Tumor tissue samples were fleetly removed for immunohistochemistry (IHC), reverse transcriptase-polymerase chain reaction (qRT-PCR), and western blot analysis. The contents of the cecum of the mice were taken and stored at − 80 °C for 16S rDNA amplicon sequencing.

### Enzyme‐linked immunosorbent assay (ELISA)

The serum levels of NLRP3, pro-caspase-1, IL-1β, IL-18, and MDA were determined using mouse NLRP3 ELISA KIT (MM-0656M1, Meimian, Wuhan, China), mouse caspase-1 ELISA KIT (MM-0820M1, Meimian), mouse pro-IL-1β ELISA KIT (MM-0905M1, Meimian), mouse pro-IL-18 ELISA KIT (MM-0906M1, Meimian), mouse IL-1β ELISA KIT (MM-0905M1, Meimian), mouse IL-18 ELISA KIT (MM-0906M1, Meimian) and mouse MDA ELISA KIT (E-EL-0060c, Elabscience, Wuhan, China) according to the manufacturer’s protocol. The absorbance of wells was determined with a microplate reader (MuLTiSKAN MK3, Thermo Fisher Scientific, Waltham, MA, USA) at 450 nm wavelength to analyze the sample concentration.

### Quantitative real-time polymerase chain reaction (qRT-PCR)

Total RNA was extracted from tumor tissue samples using TRIzol reagent (TaKaRa Biotechnology Co., Ltd., Dalian, China) according to the manufacturer's specifications. cDNA was synthesized with a PrimeScript RT reagent Kit (Takara, RR047A) in line with the manufacturer's instruction. qRT-PCR was carried out by the Bio-Rad ScripTM cDNA Synthesis Kit (Bio-Rad Laboratories, Inc., Hercules, CA, USA). The primer sequences of NLRP3 (Forward primer: 5′-ATTACCCGCCCGAGAAAGG-3′, Reverse primer: 5′-TCGCAGCAAAGATCCACACAG-3′), ASC (Forward primer: 5′-CTTGTCAGGGGATGAACTCAAAA-3′, Reverse primer: 5′-GCCATACGACTCCAGATAGTAGC-3′), and GAPDH (Forward primer: 5′-TATCGGACGCCTGGTTAC-3′, Reverse primer: 5′-CGTTCAAGTTGCCGTGTC-3′) were designed and synthesized. The qRT-PCR amplification conditions were as follows: 95 °C for 5 min, 95 °C for 15 s, and 60 °C for 30 s of 40 cycles. GAPDH was the internal control. The level of genes was analyzed by the comparative threshold cycle method (2^−△△CT^ method), where ΔΔCT = ΔCT_treatment_ −  ΔCT_control_ and ΔCT = Ct _target_ − Ct _reference_.GAPDH was used as an internal control.

### Immunohistochemistry (IHC)

Tumor samples were fixed with 4% paraformaldehyde at room temperature for 24 h. Then, paraffin sections (5 μm) were collected for the IHC assay. Sections were stained by NLRP3 (ab214185, Abcam, Cambridge, UK), ASC (67824s, Cell Signaling Technology, Inc., Danvers, MA, USA), IL-1β (2242s, CST), IL-18 (ab71495, Abcam), MDA (LS-Bio 188950, LS-Bio, Shanghai China), pro-casepase-1 (ab179515, Abcam), PKC-α (ab32376, Abcam) and BCL2L1 (A00181, Boster, Wuhan, China) antibody, respectively.

### Western blot assay

Protein samples from tumor tissue samples were obtained using a Total Protein Extraction Kit (BC3711, Solarbio). Then, the protein concentration was determined by a Protein Assay kit (Beyotime, Shanghai, China). Protein samples were separated by 10% SDS-PAGE gel and electrically transferred to PVDF membranes (Millipore, Billerica, MA, USA). The membranes were hatched with the primary antibodies at 4 ℃ overnight after being blocked with 3% bovine serum albumin (BSA, Sangon Biotech, Shanghai, China) for 1 h at room temperature. The membranes were incubated with goat-anti-rabbit IgG (H + L)-HRP (1:10,000, ab6721, Abcam) for 1 h at room temperature after washing with TBST thrice. Protein bands were visualized with an Electrochemiluminescence (ECL) chemiluminescence kit (WBULS0500; EMD Millipore) and the band’s intensity was analyzed with Image-Pro Plus 6.0 software.

### Sample DNA extraction and 16S rDNA amplicon sequencing

DNA from feces samples was extracted using PowerSoil@ DNA Isolation Kit (Bio-Tek, Vermont, USA) and qualified by 1% agarose gel electrophoresis. Then, the PCR amplification on the V3-V4 region of the sample 16S rDNA was performed with the universal primer 338F (5'-ACTCCTACGGGAGGCAGCAG-3') and 806R (5'-GGACTACHVGGGTWTCTAAT-3') to construct a PE amplification library. Sequencing was performed using the Illumina Miseq PE 2500 platform (Shanghai Zhongke New Life Biotechnology Co., Ltd., Shanghai, China). The paired-end sequencing data obtained by Miseq sequencing were removed barcode and primer splicing and further removed chimera and short sequences were to obtain clean tags. Under the condition of 97% similarity, operational taxonomic units (OTUs) clustering and species annotations were performed using qiime (v1.8.0) software. The species classification information and the annotation information of each level of each sample were obtained after compared to the representative sequence of OUTs with the corresponding microbial database. In addition, the diversity analysis based on the clustering results and the species composition information of each classification level based on the annotation results was obtained.

### Data processing and analysis

Data were shown as the means ± standard deviation. Differences among multiple groups were analyzed using one-way analysis of variance and Duncan's test using the SPSS 20.0 package. The differences were regarded as statistically non-significant and significant when p > 0.05 and p < 0.05, respectively.

## Results

### BFXJY treatment inhibited tumor growth in lung cancer with Qi-yin deficiency

**Fig. 1:**
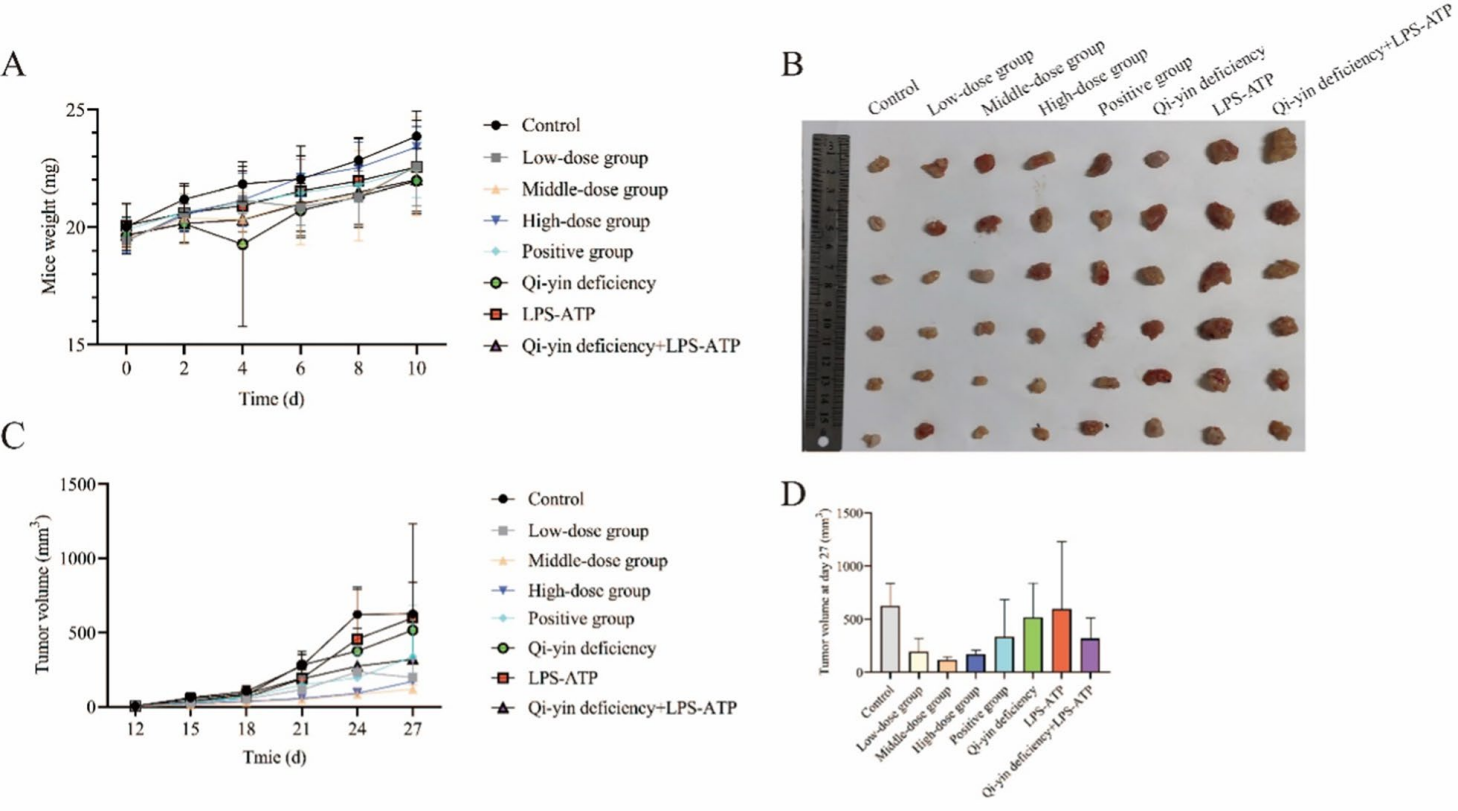
The mice weight and tumor volume. The mice’s weight (**A**) and tumor volume (**B**–**D**) were determined after mice were inoculated with A549 cells with different interventions. The means ± SD of six (tumor volume) or eight (mice weight) independent samples were shown. Differences among multiple groups were analyzed by one-way analysis of variance and followed Duncan's test using the SPSS 20.0 package. *p < 0.05 compared to the control group

After mice were inoculated with A549 cells with different interventions, the mice’s weight and tumor volume were determined. The results showed that the mice’s weight was gradually increased from day 0 to day 10, but no statistical difference was observed among all groups at day 10 (Fig. 1A). Furthermore, the tumor volume was gradually increased from day 12 to day 27, and BFXJY treatment decreased the tumor volume in control, Qi-yin deficiency, LPS-ATP, and Qi-yin deficiency + LPS-ATP groups at day 27 though no statistical difference was observed (Fig. 1B–D).

### BFXJY treatment reduced the production of IL-1β and IL-18 in lung cancer with Qi-yin deficiency

**Fig. 2:**
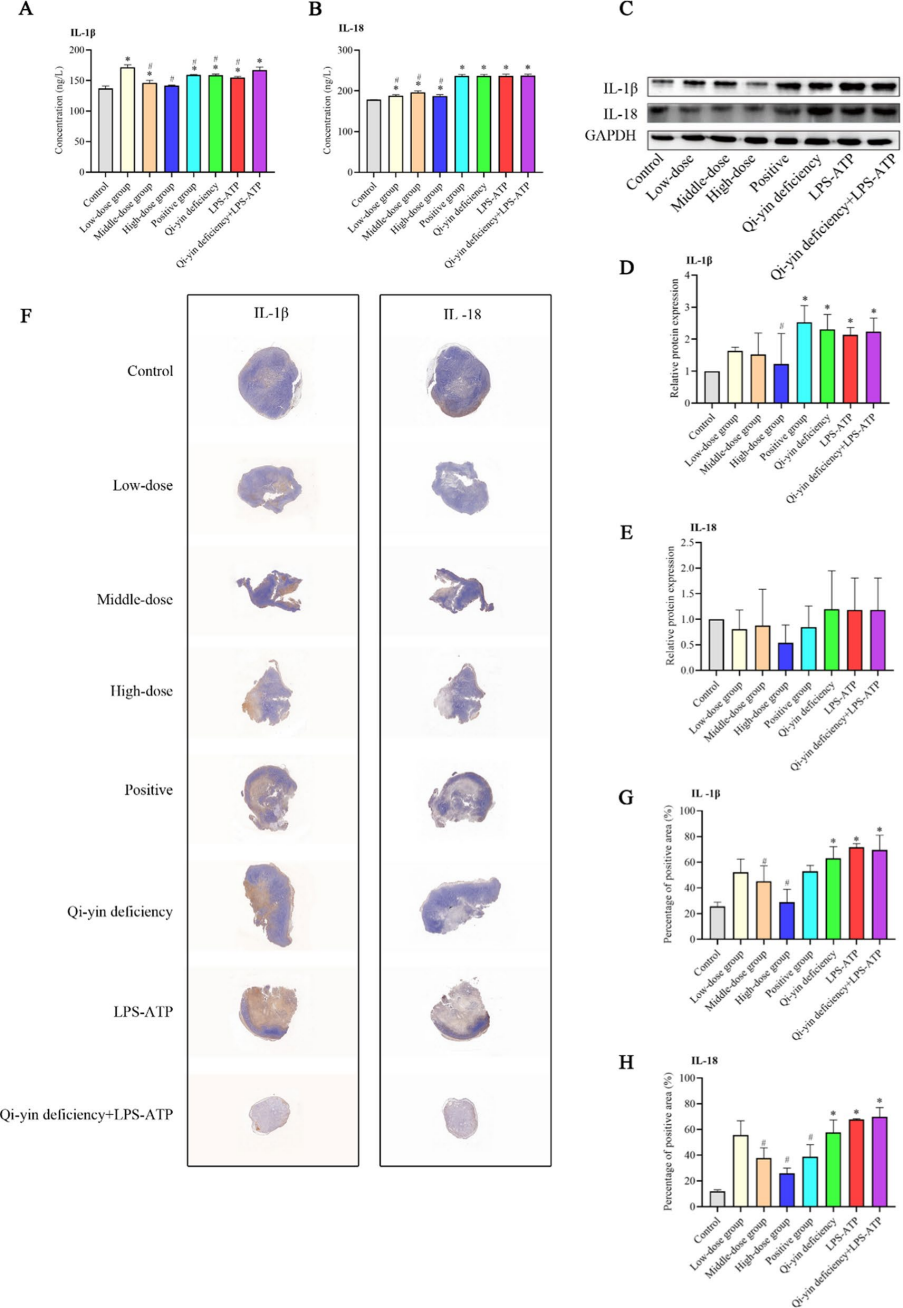
BFXJY treatment reduced the production of IL-1β and IL-18 in lung cancer with Qi-yin deficiency. **A**, **B** The serum level of IL-1β (**A**) and IL-18 (**B**) was detected using ELISA assay in lung cancer with Qi-yin deficiency. **C**–**E** The relative protein level of IL-1β (**C** and **D**) and IL-18 (**C** and **E**) was examined using western blot in lung cancer with Qi-yin deficiency. The data were expressed after being normalized to GAPDH. **F**, **G** The distribution of IL-1β (**F** and **G**) and IL-18 (**F** and **H**) was also evaluated by IHC. The means ± SD of three independent samples were shown. Differences among multiple groups were analyzed using one-way analysis of variance and followed Duncan's test using the SPSS 20.0 package. *p < 0.05 compared to the control group and ^#^*p* < 0.05 compared to the Qi-yin deficiency + LPS-ATP group

To explore the role of NLRP3 inflammasome responses in lung cancer with Qi-yin deficiency, we first detected the serum levels of IL-1β and IL-18. As shown in Fig. 2A and B, the results showed that the serum level of IL-1β and IL-18 in Qi-yin deficiency, LPS-ATP, and Qi-yin deficiency + LPS-ATP groups was significantly increased compared to that in the control group. Moreover, the serum level of IL-1β in the Qi-yin deficiency + LPS-ATP group prominently enhanced compared to that in the Qi-yin deficiency or LPS-ATP group. However, BFXJY treatment observably decreased the serum levels of IL-1β and IL-18. Similarly, the relative protein levels of IL-1β in Qi-yin deficiency, LPS-ATP, and Qi-yin deficiency + LPS-ATP groups were markedly elevated, but only the high dose BFXJY treatment markedly reduced the IL-1β relative protein level compared to that in Qi-yin deficiency + LPS-ATP group (Fig. 2C and D). No statistical difference was shown in the IL-18 relative protein expression among all groups (Fig. 2C and E). Besides, the distribution of IL-1β and IL-18 was also evaluated by IHC (Fig. 2F–H) and showed similar results within WB and ELISA. Therefore, these results indicated that BFXJY treatment reduced the production of IL-1β and IL-18 in lung cancer with Qi-yin deficiency.

### BFXJY treatment inhibited NLRP3 inflammasome activation in lung cancer with Qi-yin deficiency

**Fig. 3:**
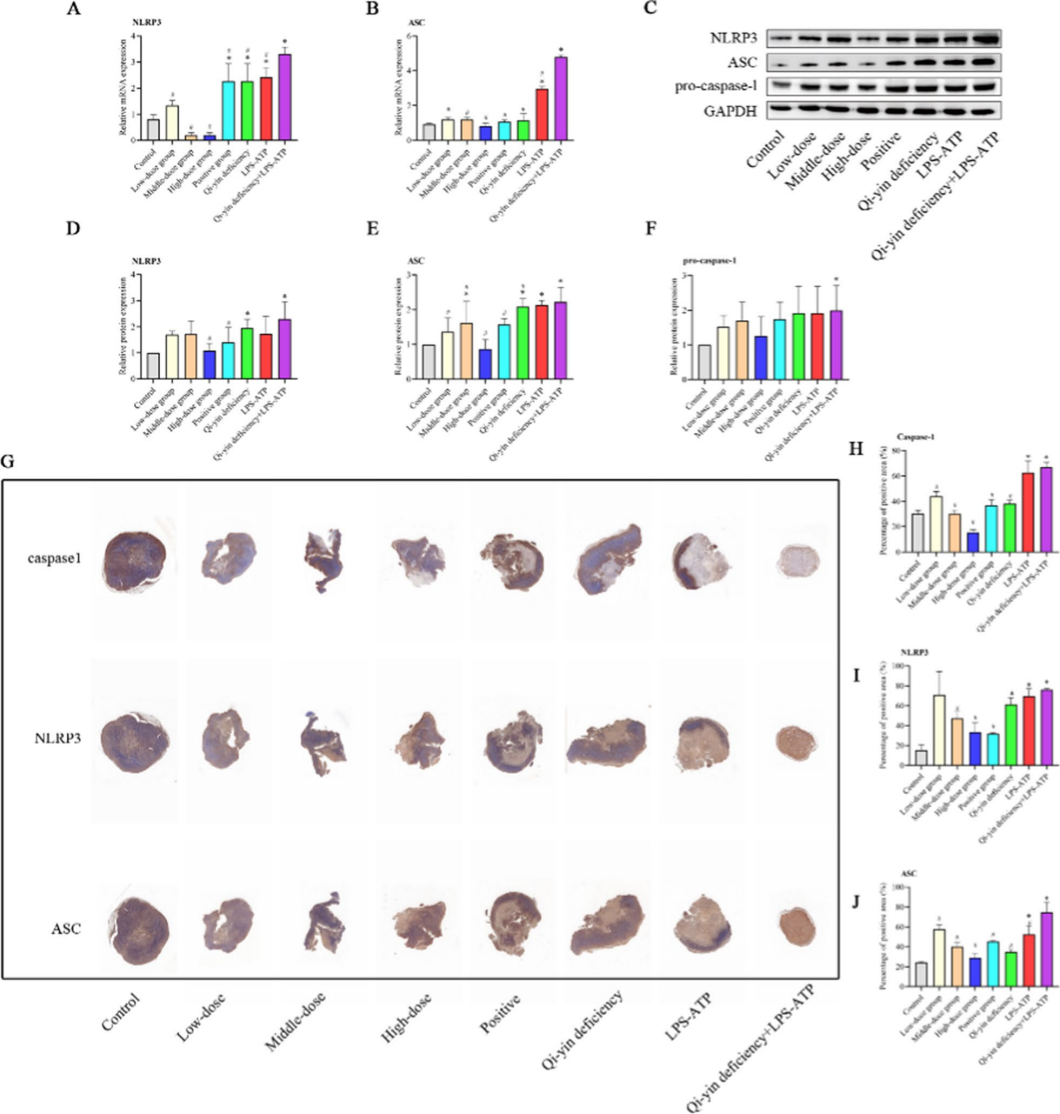
BFXJY treatment inhibited NLRP3 inflammasome activation in lung cancer with Qi-yin deficiency. **A**, **B** The relative mRNA expression of NLRP3 (**A**) and ASC (**B**) was determined using qRT-PCR. The data were expressed after being normalized to GAPDH. **C**–**F** The relative protein levels of NLRP3 (**C** and **D**), ASC (**C** and **E**), and pro-caspase-1 (**C** and **F**) were examined using western blot. The data were expressed after being normalized to GAPDH. **G**–**J** The distribution of NLRP3, ASC, and caspase-1 was also evaluated by IHC. The means ± SD of three independent samples were shown. Differences among multiple groups were analyzed by one-way analysis of variance and followed Duncan's test using the SPSS 20.0 package. *p < 0.05 compared to control group and ^#^*p* < 0.05 compared to the Qi-yin deficiency + LPS-ATP group

Additionally, NLRP3 inflammasome activation was estimated by detecting the expression of NLRP3, ASC, pro-caspase-1. In the mRNA expression level (Fig. 3A and B) and protein expression level (Fig. 3C–F), the expressions of NLRP3, ASC, and pro-Caspase-1 in Qi-yin deficiency, LPS-ATP, and Qi-yin deficiency + LPS-ATP groups were significantly increased compared to that in the control group. Moreover, the expression level of NLRP3, ASC, and pro-caspase-1 in the Qi-yin deficiency + LPS-ATP group prominently enhanced compared to that in Qi-yin deficiency or LPS-ATP group. After the treatments, the BFXJY treatment could decrease the expression levels of NLRP3, ASC, and pro-Caspase-1 compared to that in Qi-yin deficiency + LPS-ATP group. Besides, the distribution of NLRP3, ASC, and caspase-1 was also evaluated by IHC (Fig. 3G–J). Taken together, these findings suggested that BFXJY treatment inhibited NLRP3 inflammasome activation in lung cancer with Qi-yin deficiency.

### BFXJY treatment inhibited the PKC signaling in lung cancer with Qi-yin deficiency

**Fig. 4:**
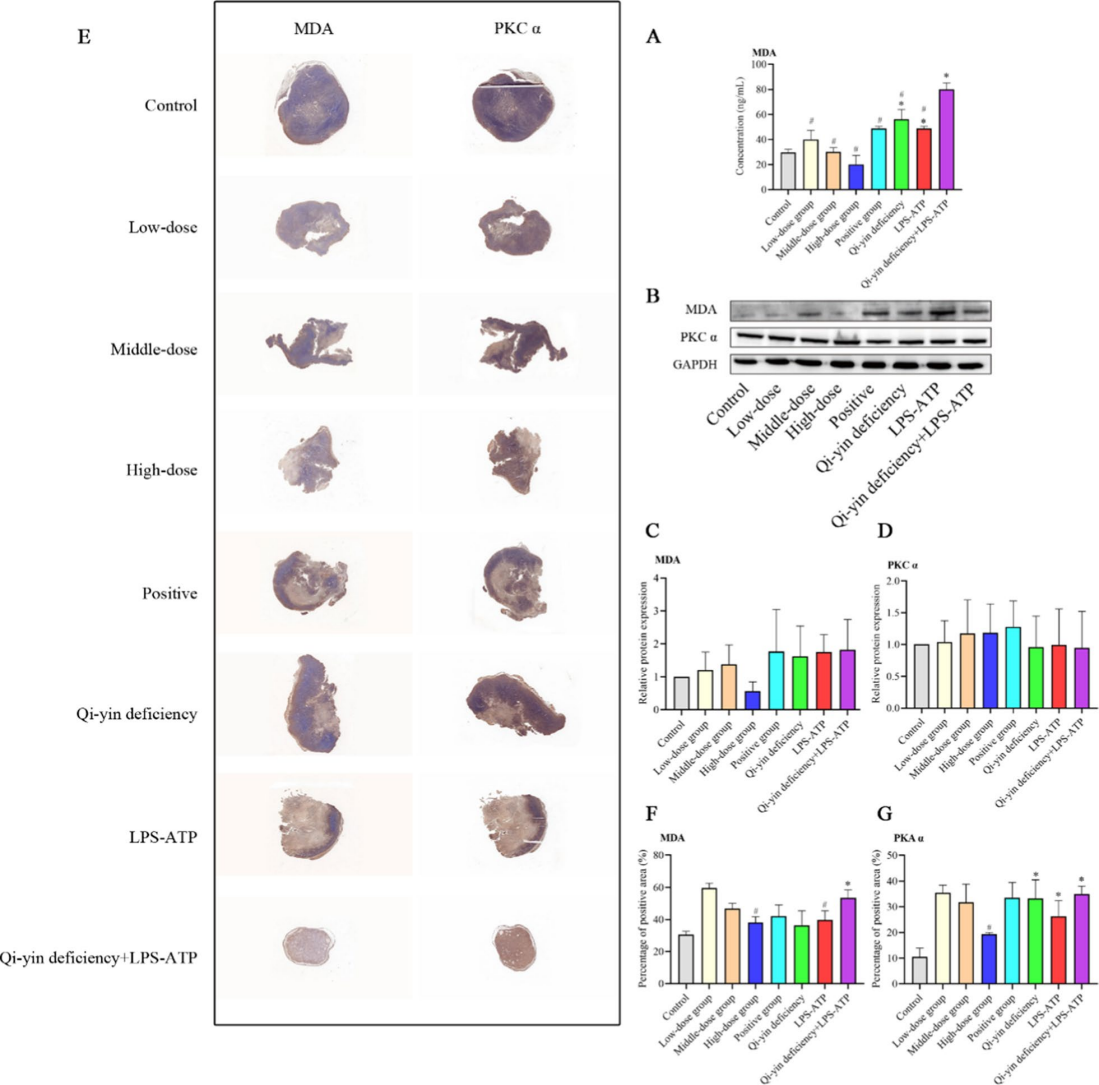
BFXJY treatment might inhibit PKC signaling in lung cancer with Qi-yin deficiency. **A** The serum level of MDA was detected using an ELISA assay. **B**–**D** The relative protein level of MDA (**B** and **C**) and PKC α (**B** and **D**) was examined using western blot in lung cancer with Qi-yin deficiency. The data were expressed after being normalized to GAPDH. **E**–**G** The distribution of MDA and PKC α was also evaluated by IHC. The means ± SD of three independent samples were shown. Differences among multiple groups were analyzed by one-way analysis of variance and followed Duncan's test using the SPSS 20.0 package. *p < 0.05 compared to control group and ^#^*p* < 0.05 compared to the Qi-yin deficiency + LPS-ATP group

It has been shown that PKC signaling is one of the signaling involved in the pro-inflammatory and pro-oxidative stress response, moreover, PKC signal can mediate oxidative stress signal to induce activation of NLRP3 inflammasome. Thus, we then assessed the role of BFXJY in PKC signaling in lung cancer with Qi-yin deficiency. The results revealed that the serum level of MDA in Qi-yin deficiency, LPS-ATP, and Qi-yin deficiency + LPS-ATP groups was significantly increased compared to that in the control group. Moreover, the serum level of MDA in the Qi-yin deficiency + LPS-ATP group prominently enhanced compared to that in the Qi-yin deficiency or LPS-ATP group. All CRID3, low, middle, and high dose BFXJY treatment markedly diminished the serum level of MDA compared to that in Qi-yin deficiency + LPS-ATP group (Fig. 4A). Although no statistical difference was shown in the MDA and PKC α relative protein expression among all groups, the protein level of MDA in low, middle, and high dose BFXJY treatment groups was declined compared with that in Qi-yin deficiency + LPS-ATP group (Fig. 4B–D). Also, the distribution of MDA and PKC α was also evaluated by IHC (Fig. 4E–G) and showed the levels of MDA and PKC α in the high dose BFXJY treatment group was declined compared with that in Qi-yin deficiency + LPS-ATP group. Therefore, all these results indicated that BFXJY treatment might inhibit PKC signaling in lung cancer with Qi-yin deficiency.

### Analysis of microbial sequence and diversity of fecal bacteria

**Fig. 5:**
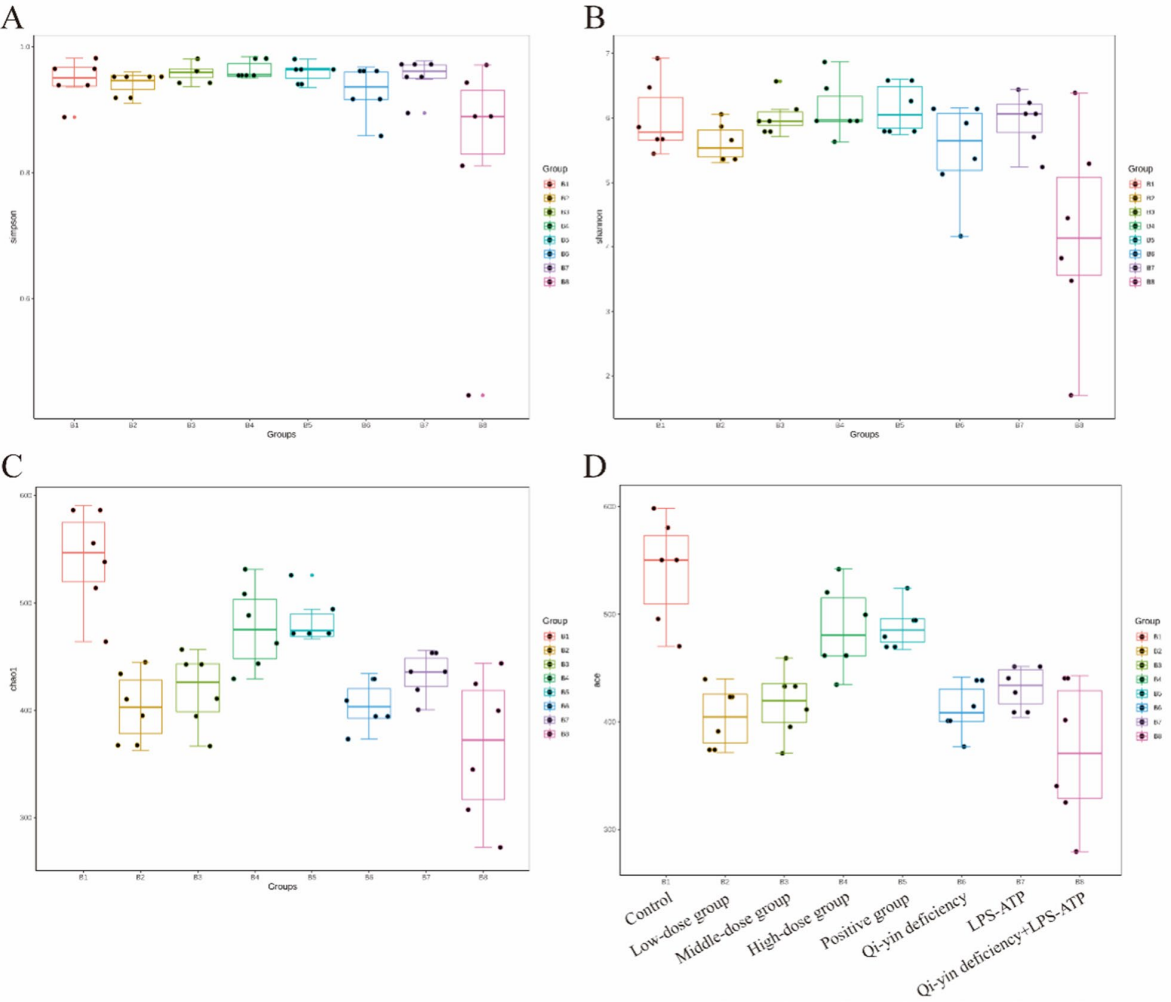
Alpha diversity index of fecal microorganisms in different intervention groups. Boxplots of bacterial alpha diversities evaluated by Simpson’s diversity index (**A**), Shannon diversity index (**B**), Chao1 index (**C**), and ACE index (**D**). Each boxplot represents the median, interquartile range, minimum, and maximum values. The means ± SD of six independent samples were shown

Six samples per group were sequenced and analyzed in the present study. After the original Tag sequences were obtained and removed out low-quality and chimera, the effective Tag sequences were used for subsequent analysis (Table 1). After all the sequences obtained from the Illumina MiSeq were clustered according to the 97% similarity of the classifiable operation units, there were 741, 593,605,730, 687, 605, 597, and 588 OTUs in control, Qi-yin deficiency, LPS-ATP, Qi-yin deficiency + LPS-ATP, Positive group, Low-dose group, Middle-dose group, and High-dose group, respectively. The common OTUs between the two groups were shown in Table 2. In addition, the Shannon and Simpson indexes in Qi-yin deficiency + LPS-ATP group was significantly lower than these in control, Qi-yin deficiency and LPS-ATP groups, while the CRID3, low, middle, and high dose BFXJY treatment, especially high dose BFXJY treatment dramatically elevated the Shannon and Simpson indexes compared to these in Qi-yin deficiency + LPS-ATP group (Fig. 5A and B). Besides, the ACE and Chao1 indexes in control, Qi-yin deficiency, and LPS-ATP groups were markedly higher than these in Qi-yin deficiency + LPS-ATP group, while the CRID3, middle and high dose BFXJY treatment, not low dose BFXJY treatment observably increased the ACE and Chao1 indexes compared to these in Qi-yin deficiency + LPS-ATP group (Fig. 5C and D). Therefore, these data suggested that the diversity of gut microbiome was reduced in lung cancer with Qi-yin deficiency. BFXJY treatment could restore the reduction that was associated with the dose of BFXJY.

**Table1:** Analysis results of quality control data

Group	Raw PE	Combined	Qualified	Q20	Q30	GC%	Effective%
Control	100,108.33	95,507.5	93,342.17	98.61	95.20	53.36	62.71
Qi-yin deficiency	104,656.67	100,071.67	97,838.5	98.64	95.36	53.73	63.87
LPS-ATP	103,748.33	101,423.83	99,261.17	98.59	95.20	53.34	63.26
Qi-yin deficiency + LPS-ATP	100,491.83	95,192.83	92,864.67	98.62	95.29	53.33	63.08
Positive group	102,530.17	100,044.83	98,036.50	98.62	95.27	53.44	62.91
Low-dose group	97,241.00	93,735.67	91,549.00	98.62	95.30	53.20	65.62
Middle-dose group	104,181.67	102,379.17	100,339.83	98.62	95.31	53.78	63.20
High-dose group	102,663.67	98,177.00	95,822.00	98.45	94.82	52.76	66.01

**Table2:** The number of OTUs

Group	OTUs	Common (vs control group)
Control	741	
Qi-yin deficiency	593	460
LPS-ATP	605	466
Qi-yin deficiency + LPS-ATP	730	489
Positive group	687	499
Low-dose group	605	459
Middle-dose group	597	466
High-dose group	588	442

### Analysis of fecal microbial composition

**Fig. 6:**
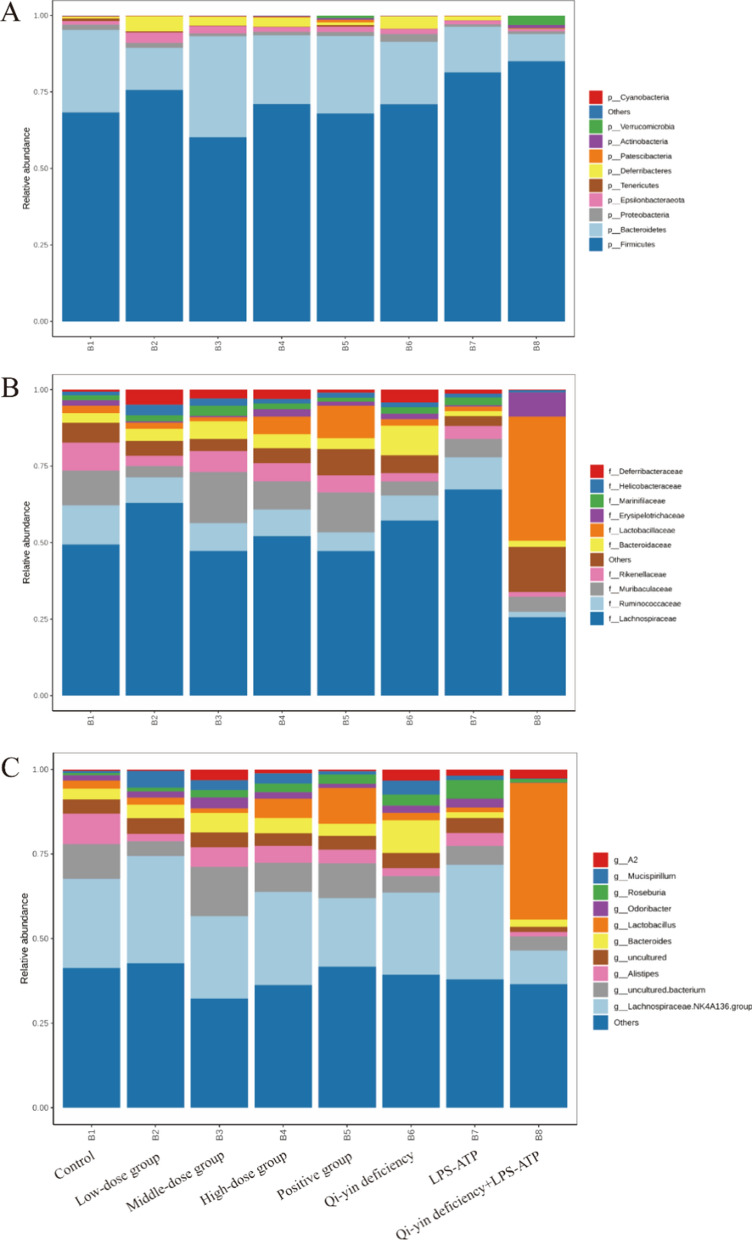
Taxonomic compositions of gut microbial communities in different levels from different intervention groups. Taxonomic distribution of gut microbial communities from each sample at the phylum level (**A**), family level (**B**) and genus level (**C**)

According to the species annotations, the top 10 species with the largest abundance at each classification level for each sample or group were selected and analyzed the relative abundance and the proportions at different classification levels. Based on the level of phylum classification (Fig. 6A), *Firmicutes* and *Bacteroidetes* were the main phyla detected in feces of each group, among which *Firmicutes* had the highest relative abundance followed by *Bacteroidetes*. Compared to those in the control group, the relative abundance of *Firmicutes* and *Verrucomicrobia* was increased, while the relative abundance of *Bacteroidetes, Proteobacteria*, and *Epsilonbacteraeota* was decreased in Qi-yin deficiency + LPS-ATP group. BFXJY treatment, especially middle dose BFXJY treatment notably reduced the Qi-yin deficiency combined with LPS-ATP induced the enhancement of the relative abundance of *Firmicutes* and *Verrucomicrobia*, while increased the Qi-yin deficiency combined with LPS-ATP induced the reduction of the relative abundance of *Bacteroidetes* and *Epsilonbacteraeota*. Besides, BFXJY treatment observably enhanced the relative abundance of Deferribacteres. Figure 6B showed the top 10 families with relative content. Compared to those in the control group, the relative abundance of *Lachnospiraceae, Ruminococcaceae, Muribaculaceae*, and *Rikenellaceae* was obviously declined, while the relative abundance of *Lactobacillaceae* and *Erysipelotrichaceae* was significantly elevated in Qi-yin deficiency + LPS-ATP group. However, BFXJY treatment distinctly reversed the change in Qi-yin deficiency + LPS-ATP group. The top 10 genera as shown in Fig. 6C. Compared to these in the control group, the relative abundance of *Lachnospiraceae NK4A136* group and *Alistipes* decreased, while the relative abundance of *Lactobacillus* and *A2* prominently increased in Qi-yin deficiency + LPS-ATP group. Similarly, BFXJY treatment overtly reversed the alteration in Qi-yin deficiency + LPS-ATP group. Overall, all these data indicated that BFXJY treatment could obviously antagonize the change caused by Qi-yin deficiency combined with LPS-ATP treatment.

## Discussion

In the present study, we have demonstrated that NLRP3 inflammasome responses were upregulated in lung cancer with Qi-yin deficiency, which could be relieved with BFXJY treatment. Moreover, the diversity of gut microbiome was reduced in lung cancer with Qi-yin deficiency, and BFXJY treatment could restore the reduction that was associated with the dose of BFXJY. Furthermore, BFXJY treatment could obviously antagonize the change of fecal microbial composition caused by Qi-yin deficiency combined with LPS-ATP treatment.

A large number of experimental and clinical evidence show that inflammation has an important role in promoting tumor development at all stages [27]. The NLRP3 inflammasome is not only the central link in the occurrence of inflammation, it can also promote tumor formation, invasion, and metastasis, and has become a research hotspot in tumor treatment [28]. After the NLRP3 inflammasome is activated, it causes an inflammatory cascade reaction, leading to the release of pro-inflammatory factors such as IL-1β, IL-18, IL-22, TNF-α, and the activation of inflammation-tumor signaling pathways such as NF-κB. The tumor microenvironment promotes the growth of tumor cells and ultimately leads to the occurrence and deterioration of tumors. In the occurrence and development of gastric cancer and colon cancer, inflammasomes have been characterized [29]. IL-1β is an important product of the NLRP3 inflammasome. Studies have shown that transgenic mice that specifically express IL-1β can recruit a large number of myeloid-derived suppressor cells at an early stage and are more likely to develop spontaneous gastritis and gastric cancer. Blocking the IL-1β signaling pathway can well inhibit the development of gastric precancerous and the activation of myeloid-derived suppressor cells [30]. However, some studies have also shown that in the enteritis model, activation of NLRP3 inflammasome has a protective effect on enteritis [31], and it has also been found that NLRP3 inflammasome can also inhibit the occurrence of enteritis-related tumors [32]. Tas et al. [33] has shown that over-expression of IL-18 promotes the occurrence of gastric cancer and affects the prognosis of patients. The level of IL-18 in serum may be an indicator for the diagnosis of gastric cancer. Moreover, a large number of studies have shown that in lung cancer with Qi-yin deficiency, IL-1β and other inflammatory factors are all upregulated, which plays an important role in promoting the development of lung cancer. For example, Shen et al. [34] found that the expression levels of IL-1β and TNF-α are closely related to lung-yin deficiency syndrome. Cai et al. [35] reported that the changes of serum TNF-α, endothelin (ET), and MDA content in rats with lung cancer with Qi-yin deficiency can be used as one of the important objective indicators of lung cancer with Qi-yin deficiency. Liang et al. [12] performed TCM differentiation of 38 patients with advanced lung cancer cachexia, and detected serum TNF-α, IL-1, and IL-6 levels, indicating that the TCM differentiation of lung cancer cachexia was mainly based on Qi-yin deficiency, and IL-6 and IL-1β levels are significantly higher than control people. Therefore, in lung cancer with Qi-yin deficiency, IL-1β and other interleukin pro-inflammatory factors are expressed in large quantities [12], which are also the main products of NLRP3 inflammasomes [36]. Previous studies have been shown that IL-1 and MDA are important indicators of Qi-yin deficiency [37]. Moreover, the previous research [11] shows that NLRP3 inflammasome and its products are up-regulated in lung cancer tissues. In line with these results, our results also revealed the up-regulation of NLRP3 inflammasome and its products, including NLRP3, ASC, IL-1β, MDA at serum level, mRNA level, and protein level.

BuFeiXiaoJiYin (BFXJY) is created by our group based on many years of clinical experience. We have used the human non-small cell lung cancer cell line A549 to explore the anti-NSCLC effect of BFXJY through MTT, flow cytometry, RT-PCR, and other experimental methods [22, 23, 38]. The results showed that BFXJY can inhibit the growth of lung cancer cells and prevent the malignant proliferation of lung cancer cells; BFXJY induces tumor cell apoptosis and increases the rate of cell apoptosis, thereby exerting anti-tumor effects; The mechanism of BFXJY inducing apoptosis of non-small cell lung cancer cells may be through reducing MGMT. The level of methylation affects the expression of other apoptosis-related factors or the conduction of related pathways. Moreover, several components, such as Mulberry Bark and Curcuma zedoary, have been exhibited well inhibitory effects on the expression of IL-1β, IL-18, caspase-1, and NLRP3-ASC, which suggested that BFXJY can exert an anti-inflammation role through NLRP3 inflammasome responses [24]. Expectedly, in the present study, we found that BFXJY treatment relieved the up-regulation of NLRP3 inflammasome responses in lung cancer with Qi-yin deficiency, as indicated by the decrease of the serum, mRNA, and protein level of NLRP3, ASC, IL-1β, MDA.

There are at least 1,000 kinds of microorganisms in the human intestine, with some up to 10 trillion. These microorganisms maintain a dynamic balance in the intestine, which can synthesize vitamins, help the body absorb nutrients from food, maintain the function of the intestinal immune system, and resist the damage of harmful microorganisms, which is called the "second genome" acquired by the human body [39]. The diversity of microbial communities is often evaluated by α diversity and β diversity. α Diversity reflects the diversity and richness of species in the sample. Specific evaluation indicators include Simpson, Shannon, Chao1, and ACE indices. Among them, Chao1 and ACE indexes are used to measure species richness, that is, the number of species; Shannon and Simpson indexes are used to measure species diversity [40]. In the present study, the Shannon and Simpson indexes in the Qi-yin deficiency + LPS-ATP group were significantly decreased, which was dramatically reversed with low, middle, and high dose BFXJY treatment. The reduced Chao1 and ACE indexes in Qi-yin deficiency + LPS-ATP group were observably increased with middle and high dose BFXJY treatment, not low dose BFXJY treatment. Thus, we concluded that the diversity of gut microbiome was reduced in lung cancer with Qi-yin deficiency, which could be antagonized with BFXJY treatment and was associated with the dose of BFXJY. Studies have found that the imbalance of the intestinal microbial community often stems from changes in the abundance of the *Proteobacteria*. Therefore, the *Proteobacteria* can be used as a microbial marker for the imbalance of the intestinal flora [41]. Consistently, our results also showed a decrease in the Qi-yin deficiency + LPS-ATP group, indicating the imbalance of the intestinal flora in the Qi-yin deficiency + LPS-ATP group. The relative abundance of *Lachnospiraceae*, *Ruminococcaceae*, *Muribaculaceae,* and *Rikenellaceae* are negatively correlated with Qi-yin deficiency combined with LPS-ATP, which was similar to a recent study Pu-erh tea ameliorates obesity and modulates gut microbiota in the high fat-diet-fed mice [42]. Several genera were also regulated in the Qi-yin deficiency + LPS-ATP group, including a decrease of *Lachnospiraceae NK4A136 group* and *Alistipes*, and an increase of *Lactobacillus* and *A2*. However, all the change in the taxonomic distribution of gut microbial communities from each sample at the phylum level, family level, and genus level were inverted with BFXJY treatment. Therefore, the diversity and microbial composition of gut microbiome were changed in lung cancer with Qi-yin deficiency, and BFXJY treatments could antagonize the alteration and were associated with the dose of BFXJY. However, it is still unclear whether the microbiota alteration is due to an indirect effect resulting from a reduced tumor or a direct regulation gut from BFXJY and this needs further study.

## Conclusion

In conclusion, our results demonstrated that NLRP3 inflammasome responses were upregulated in lung cancer with Qi-yin deficiency, which could be relieved with BFXJY treatment. Moreover, the diversity of gut microbiome was reduced in the lung cancer with Qi-yin deficiency, and BFXJY treatment could restore the reduction that was associated with the dose of BFXJY. Furthermore, BFXJY treatment could obviously antagonize the change of fecal microbial composition caused by Qi-yin deficiency combined with LPS-ATP treatment. Taken together, the results provide a theoretical basis for the clinical development of therapeutic drugs targeting to treat lung cancer.

## Data Availability

The datasets used and/or analyzed during the current study are available from the corresponding author on reasonable request.

## Abbreviations

NSCLC: Non‐small cell lung cancer
NOD: Nucleotide-binding oligomerization domain
NLRP3: Nucleotide-binding oligomerization domain-like receptor family, pyrin domain-containing protein 3
ASC: A caspase activation and recruitment domain
PAMPs: Pathogen-associated molecular patterns
DAMPs: Damage-associated molecular patterns
LPS: Lipopolysaccharide
ATP: Adenosine triphosphate
CRC: Colorectal cancer
MALT: Mucosa-associated lymphoid tissue
OTUs: Operational taxonomic unitse

## References

[1] Siegel RL, Miller KD, Jemal A (2018). Cancer statistics, 2018. CA Cancer J Clin.

[2] Hashim D, Boffetta P, La Vecchia C (2016). The global decrease in cancer mortality: trends and disparities. Ann Oncol.

[3] Liu X, Wu S, Yang Y (2017). The prognostic landscape of tumor-infiltrating immune cell and immunomodulators in lung cancer. Biomed Pharmacother.

[4] Balkwill F, Coussens LM (2004). Cancer: an inflammatory link. Nature.

[5] Schreiber RD, Old LJ, Smyth MJ (2011). Cancer immunoediting: integrating immunity's roles in cancer suppression and promotion. Science.

[6] Pinkerton JW, Kim RY, Robertson AAB (2017). Inflammasomes in the lung. Mol Immunol.

[7] Guo H, Callaway JB, Ting JP (2015). Inflammasomes: mechanism of action, role in disease, and therapeutics. Nat Med.

[8] Dostert C, Pétrilli V, Van Bruggen R (2008). Innate immune activation through Nalp3 inflammasome sensing of asbestos and silica. Science.

[9] Schroder K, Tschopp J (2010). The inflammasomes. Cell.

[10] Agostini L, Martinon F, Burns K (2004). NALP3 forms an IL-1beta-processing inflammasome with increased activity in Muckle-Wells autoinflammatory disorder. Immunity.

[11] Kong H, Wang Y, Zeng X (2015). Differential expression of inflammasomes in lung cancer cell lines and tissues. Tumour Biol.

[12] Liang C, Yang B, Du J (2010). The relationship between cytokines and syndrome differentiation of traditional Chinese medicine in lung cancer induced cachexia. J Anhui Tradit Chin Med Coll.

[13] Donovan C, Liu G, Shen S (2020). The role of the microbiome and the NLRP3 inflammasome in the gut and lung. J Leukoc Biol.

[14] Cottier F, Pavelka N (2012). Complexity and dynamics of host-fungal interactions. Immunol Res.

[15] Purchiaroni F, Tortora A, Gabrielli M (2013). The role of intestinal microbiota and the immune system. Eur Rev Med Pharmacol Sci.

[16] Feng Y, Huang Y, Wang Y (2019). Antibiotics induced intestinal tight junction barrier dysfunction is associated with microbiota dysbiosis, activated NLRP3 inflammasome and autophagy. PLoS ONE.

[17] Lowe PP, Gyongyosi B, Satishchandran A (2018). Reduced gut microbiome protects from alcohol-induced neuroinflammation and alters intestinal and brain inflammasome expression. J Neuroinflamm.

[18] Fernández MF, Reina-Pérez I, Astorga JM (2018). Breast cancer and its relationship with the microbiota. Int J Environ Res Public Health.

[19] Raza MH, Gul K, Arshad A (2019). Microbiota in cancer development and treatment. J Cancer Res Clin Oncol.

[20] Gui QF, Lu HF, Zhang CX (2015). Well-balanced commensal microbiota contributes to anti-cancer response in a lung cancer mouse model. Genet Mol Res.

[21] Zhang WQ, Zhao SK, Luo JW (2018). Alterations of fecal bacterial communities in patients with lung cancer. Am J Transl Res.

[22] Rong Z, Wei H, Chen X (2014). Clinical investigation of Bufei Xiaoji decoction in treatment of patients with advanced drugs-resistant non-small cell lung cancer. Chin J Exp Tradit Med Formulae.

[23] Rong Z, Huang Y, Mo C (2013). Effect of Bufei Xiaoji decoction combined with chemotherapy on immune escape of lung cancer. Tianjin Med J.

[24] Yuvaraj K, Geetha A (2018). Effect of Morus alba root bark extract on gene-level expression of inflammatory markers in rats subjected to ethanol and cerulein induced pancreatitis- influence of heat shock protein 70. J Complement Integr Med.

[25] Xing S, Wang Y, Hu K (2020). WGCNA reveals key gene modules regulated by the combined treatment of colon cancer with PHY906 and CPT11. Biosci Rep.

[26] Li S, He Y, Zhang H (2020). Formulation of traditional Chinese medicine and its application on intestinal flora of constipated rats. Microb Cell Fact.

[27] Zuo H, Tell GS, Vollset SE (2014). Interferon-γ-induced inflammatory markers and the risk of cancer: the Hordaland Health Study. Cancer.

[28] Müzes G, Sipos F (2015). Inflammasome, inflammation and cancer: an interrelated pathobiological triad. Curr Drug Targets.

[29] Kolb R, Liu GH, Janowski AM (2014). Inflammasomes in cancer: a double-edged sword. Protein Cell.

[30] Tu S, Bhagat G, Cui G (2008). Overexpression of interleukin-1beta induces gastric inflammation and cancer and mobilizes myeloid-derived suppressor cells in mice. Cancer Cell.

[31] Zaki MH, Boyd KL, Vogel P (2010). The NLRP3 inflammasome protects against loss of epithelial integrity and mortality during experimental colitis. Immunity.

[32] Allen IC, TeKippe EM, Woodford RM (2010). The NLRP3 inflammasome functions as a negative regulator of tumorigenesis during colitis-associated cancer. J Exp Med.

[33] Tas F, Tilgen Yasasever C, Karabulut S (2015). Clinical significance of serum interleukin-18 (IL-18) levels in patients with gastric cancer. Biomed Pharmacother.

[34] Shen W, Sun Y, Zhang S, Yu G (2000). Study on the correlation between interleukin-1 and the essence of Lung Yin deficiency syndrome. J Tradit Chin Med.

[35] Zhao S, Cai S, Fang Z, Huang K, Wang Y (2003). Study on the changes and correlation of blood gas analysis index with thromboxane and endothelin in lung Qi deficiency rats. Chin J Basic Med Tradit Chin Med.

[36] De Nardo D, De Nardo CM, Latz E (2014). New insights into mechanisms controlling the NLRP3 inflammasome and its role in lung disease. Am J Pathol.

[37] Wu H, Tian J (2012). Establishment and evaluation of animal model of lung cancer with deficiency of qi and Yin. Zhejiang J Integr Med.

[38] Rong Z, Xu Y, Mo C, Lian Z, Chen X (2012). Effects of Dujieqing Oral liquid on the promoter methylation of the MGMT gene in middle-and-late stage tumor patients receiving chemotherapy. Chin J Integr Tradit Western Med.

[39] Adak A, Khan MR (2019). An insight into gut microbiota and its functionalities. Cell Mol Life Sci.

[40] Grice EA, Kong HH, Conlan S (2009). Topographical and temporal diversity of the human skin microbiome. Science.

[41] Boursi B, Mamtani R, Haynes K (2015). Recurrent antibiotic exposure may promote cancer formation–another step in understanding the role of the human microbiota?. Eur J Cancer.

[42] Ye J, Zhao Y, Chen X (2021). Pu-erh tea ameliorates obesity and modulates gut microbiota in high fat diet fed mice. Food Res Int.

